# Two-dimensional CT measurements enable assessment of body composition on head and neck CT

**DOI:** 10.1007/s00330-022-08773-9

**Published:** 2022-04-07

**Authors:** David Zopfs, Daniel Pinto dos Santos, Jonathan Kottlors, Robert P. Reimer, Simon Lennartz, Roman Kloeckner, Max Schlaak, Sebastian Theurich, Christoph Kabbasch, Marc Schlamann, Nils Große Hokamp

**Affiliations:** 1 Institute for Diagnostic and Interventional Radiology, Faculty of Medicine and University Hospital Cologne, University of Cologne, Kerpener Str. 62, 50937 Cologne, Germany; 2 Institute for Diagnostic and Interventional Radiology, University Hospital Frankfurt, Frankfurt am Main, Germany; 3 Department of Diagnostic and Interventional Radiology, University Medical Center of the Johannes Gutenberg University Mainz, Mainz, Germany; 4 Department of Dermatology, Venerology and Allergology, Charité - Universitätsmedizin Berlin, Corporate Member of Freie Universität Berlin, Humboldt-Universität, Berlin, Germany; 5 Cancer- and Immunometabolism Research Group, Gene Center LMU, Ludwig Maximilian University, Munich, Germany; 6 Department of Medicine III, University Hospital LMU, Ludwig Maximilian University, Munich, Germany

**Keywords:** Sarcopenia, Intra-abdominal fat, Tomography, X-ray computed, Body composition, Electrical impedance

## Abstract

**Objectives:**

The aim of this study was to evaluate whether simple 2D measurements in axial slices of head and neck CT examinations correlate with generally established measurements of body composition in abdominal CT at the height of the third lumbar vertebra and thus allow for an estimation of muscle and fat masses.

**Methods:**

One hundred twenty-two patients who underwent concurrent CT of the head and neck and the abdomen between July 2016 and July 2020 were retrospectively included. For a subset of 30 patients, additional bioelectrical impedance analysis (BIA) was available. Areas of paraspinal muscles at the height of the third (C3) and fifth cervical vertebrae (C5) as well as the total cross-sectional area at the height of C3 and at the submandibular level were correlated with the results of abdominal measurements and BIA. Furthermore, intra- and interreader variabilities of all measurements were assessed.

**Results:**

Regarding adipose tissue, good correlations were found between the total cross-sectional area of the patient’s body at the submandibular level and at the height of C3 between both abdominal measurements and BIA results (*r* = 0.8–0.92; all *p* < 0.001). Regarding muscle, the total paraspinal muscle area at the height of C3 and C5 showed strong correlations with abdominal measurements and moderate to strong correlations with BIA results (*r* = 0.44–0.80; all *p* < 0.001), with the muscle area on C5 yielding slightly higher correlations.

**Conclusions:**

Body composition information can be obtained with comparable reliability from head and neck CT using simple biplanar measurements as from abdominal CT.

**Key Points:**

• *The total paraspinal muscle area at the height of C3 and C5 correlates strongly with abdominal muscle mass.*

• *The total cross-sectional area at the submandibular level and at the height of C3 shows good correlations with abdominal fat mass.*

• *The described measurements facilitate a rapid, opportunistic assessment of relevant body composition parameters.*

**Supplementary Information:**

The online version contains supplementary material available at 10.1007/s00330-022-08773-9.

## Introduction

Body composition and its pathological changes, such as sarcopenia, loss of muscle mass, and sarcopenic obesity, are established prognostic markers in various diseases. Their influence has been evaluated in different clinical scenarios ranging from oncological diseases to postoperative courses following several surgical procedures and cardiovascular and metabolic diseases as well as emergency settings [1–4].

Since chronically ill patients usually undergo radiological imaging on a regular basis, it seems appealing to obtain quantitative body composition information from clinically indicated cross-sectional imaging opportunistically. This offers the advantage of eliminating the necessity for patients to receive additional, dedicated examinations, which assess muscle and adipose tissue, such as dual-energy X-ray absorptiometry (DEXA) or bioelectrical impedance analysis (BIA). Imaging-based assessment of body composition is usually performed in abdominal cross-sectional examinations at the height of the third lumbar vertebra (L3) [5, 6]. Here, numerous studies have shown that results of simple 2D measurements and more elaborate segmentations as well as results of fully automated approaches are valid biomarkers of body composition [6–10].

However, many patients with neurological diseases receive only cross-sectional imaging of the head or the head and neck, hampering the opportunistic acquisition of body composition based on well-established abdominal parameters. Accordingly, studies on the influence of body composition on neurological diseases are relatively sparse. For example, there are only a very few studies that have investigated the relationship between body composition information obtained opportunistically from imaging data and outcome in patients with stroke, although this is a common and serious disease [11]. In contrast, there is much more data on the influence of body composition in abdominal diseases. This could be due, in part, to the fact that there have been few simple and validated measurements outside of abdominal imaging.

While a few studies have also demonstrated the possibility of obtaining reliable body composition information at the level of the chest, the data on the feasibility of obtaining data in patients with neck-only examinations is scarce [12–14]. A sporadic number of studies outlined that muscle areas measured at the level of the third cervical vertebra (C3) correlate with muscle areas measured at the level of L3 [15–17]. However, the approaches described so far to obtain body composition information from cervical CT scans either are relatively laborious, require additional clinical parameters, or cannot be obtained from visual muscle delineations alone, but require special preparations such as additional measurements of densities [15, 16]. Therefore, demand remains for a straightforward, easy-to-perform assessment of body composition in head and neck CT.

Thus, the aim of this study was to evaluate straightforward biplanar measurements in axial CT images of the neck with respect to their potential usefulness for body composition assessment and to compare them with well-established measurements at the level of L3 and non-imaging body composition information derived from BIA.

## Materials and methods

### Patient collective

Our retrospective, monocentric study was approved by the institutional review board. The requirement for informed consent was waived. A merged query to the radiological information system and the picture archiving and communication system was performed to identify adult patients, who were referred from the Department for Dermatology for CT-staging examination of the neck, chest, and abdomen between June 2016 and July 2020. For a subset of 30 patients, additional BIA body composition analysis was available in the context of a different, independent prospective study, which was approved by the institutional review board (No. 16-239, University of Cologne). For the subset of these 30 patients, written informed consent regarding BIA assessment was obtained from every patient.

### Imaging protocol

All patients underwent CT-staging examinations as part of the standard clinical care on a 64-row spectral detector CT (IQon, Philips Healthcare). Scanning was performed in a head-first, supine patient position, with the head supported by a head cushion. Every patient was examined using a standardized examination protocol, which comprised a portal venous phase acquisition of the chest and abdomen, followed by a separated venous phase scan of the neck. The following scan settings were employed for the chest and abdomen examination: tube current modulation (DoseRight 3D-DOM, Philips Healthcare), tube voltage 120 kVp, rotation time 0.33 s, pitch 0.671, collimation 64 × 0.625 mm, and a matrix of 512 × 512. For the neck examination, the following scan settings were used: tube current modulation (DoseRight 3D-DOM, Philips Healthcare), tube voltage 120 kVp, rotation time 0.5 s, pitch 0.985, collimation 64 × 0.625, and a matrix of 512 × 512. Scanning parameters resulted in a CTDI_vol_ of 11.7 mGy for scans of the neck and in a CTDI_vol_ of 11.5 mGy for scans of the chest and abdomen. A total of 180 ml of iodinated contrast media (Accupaque, 350 mg/ml; GE Healthcare) is routinely administered as a bolus using a 20-G intravenous catheter. Here, 100 ml is allocated to the chest and abdomen scan and 80 ml to the subsequent neck scan. Images were reconstructed with a slice thickness of 2 mm and a 1-mm section increment using a hybrid iterative reconstruction algorithm (iDose 4, filter B, level 3, Philips Healthcare).

### CT measurements

Reading was carried out by two radiologists with 4 and 5 years of experience. To determine intrareader reliability, one reader re-performed all measurements after 4 weeks to avoid recall bias. All 2D measurements were performed using a standard DICOM viewer (Impax EE R20, XVII SU1, Agfa Healthcare). Regions of interest (ROI) were created to determine muscle and total cross-sectional areas using a freehand annotation embedded in the viewing application.

**Fig. 1:**
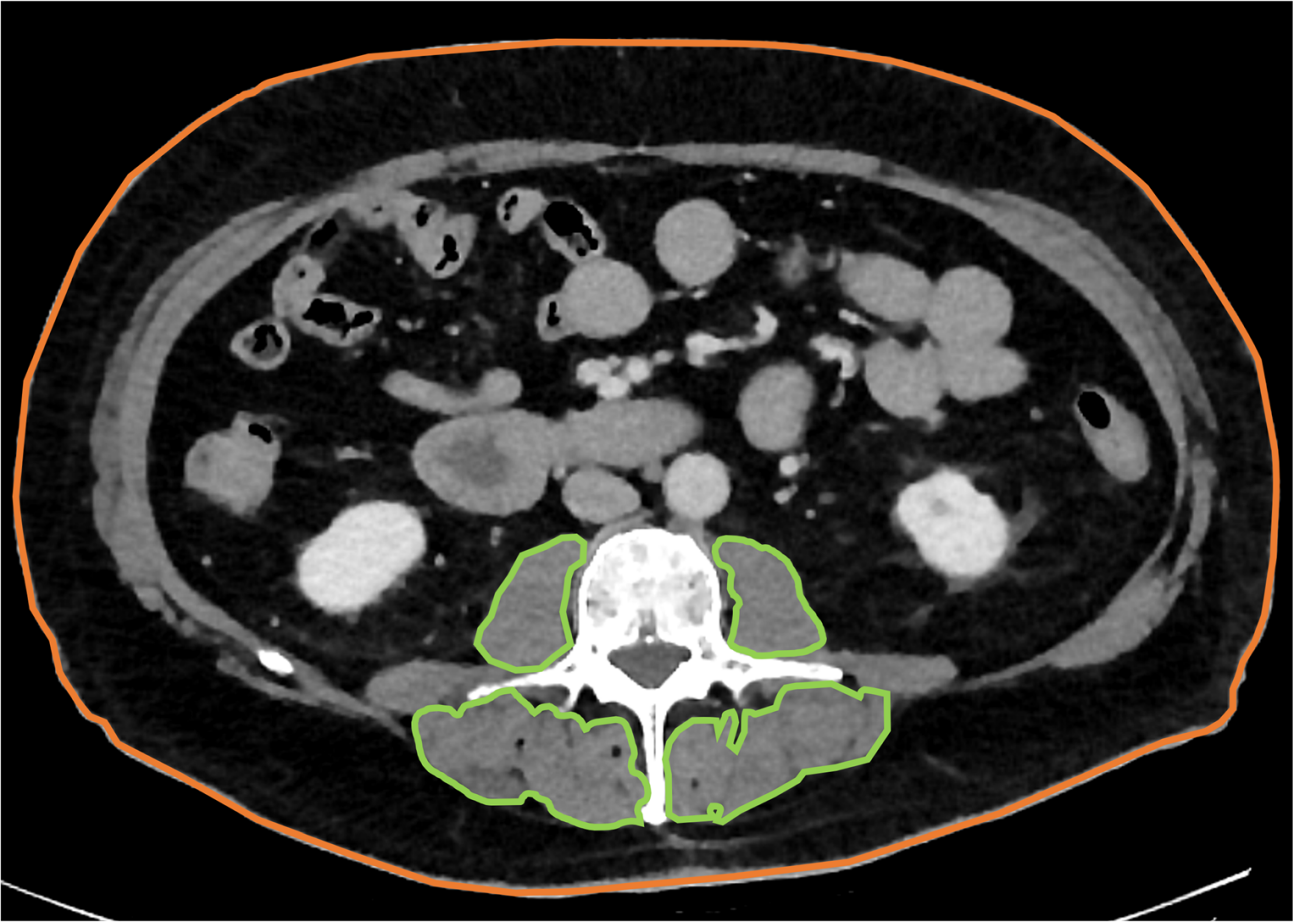
Overview of the abdominal 2D measurements. Measurements of muscle (outlined in green) and the total cross-sectional area (outlined in orange) were performed using a freehand ROI tool within the standard PACS at the height of the third lumbar vertebra

The abdominal measurements were carried out at the height of L3 in accordance with earlier, well-established methods: A sagittal plane was used to identify L3, and all measurements were then performed on an axial plane located centrally at this height. The area of the following regions was recorded: psoas major muscles, autochthonous spine muscles, and total cross-sectional area of the patient’s body. The combined total of all individual muscle areas was defined as the total abdominal paraspinal muscle area (see Fig. 1).

**Fig. 2:**
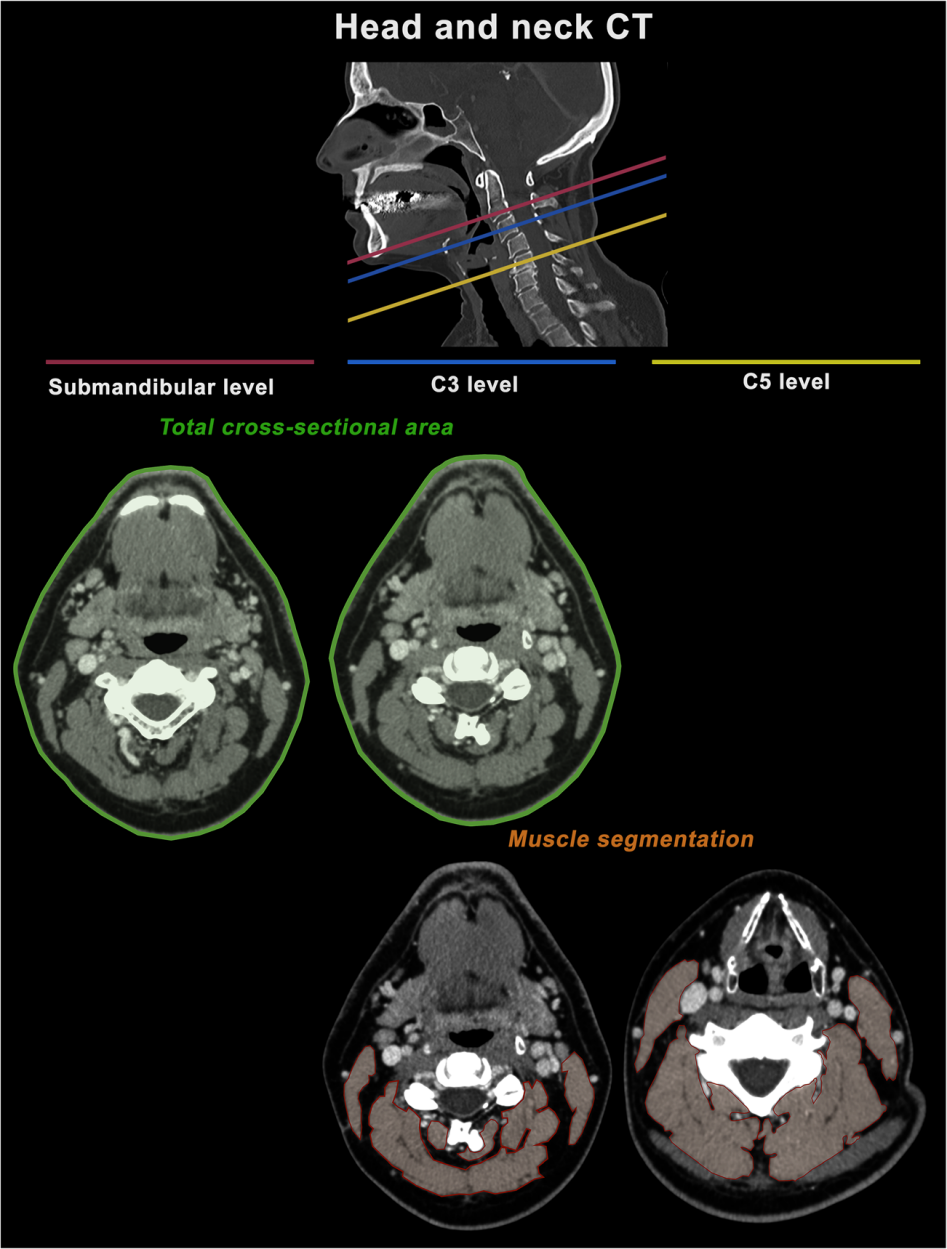
Overview of the cervical 2D measurements to assess body composition. All measurements were performed using a freehand ROI tool within the standard PACS. The total cross-sectional area was measured at the submandibular level and at height of the third cervical vertebra (C3, outlined in green). Paraspinal muscle areas were measured at the height of C3 and at the height of the fifth cervical vertebra (C5, outlined in orange)

At the level of the neck, the total cross-sectional area of the patient’s body was measured at the height of C3 as well as submandibular. Furthermore, muscle areas of the sternocleidomastoid muscle and the autochthonous muscles were assessed at the height of the third and the fifth cervical vertebrae (C5). If the trapezius muscle was depicted at the level of C5, it was not considered. Analogously, the sum of all muscle areas was defined as the total cervical paraspinal muscle area at the height of C3 and C5, respectively. During ROI placement, small vessels or nerves within the muscles were included in measurements, as their contribution to the overall area appeared negligible. Figure 2 illustrates all cervical measurements.

### Bioelectrical impedance analyses

BIA was conducted using a multi-frequency BIA apparatus (Seca mBCA 515, Seca) in overnight-fasted patients during the morning. Tetrapolar measurements of bioelectrical impedance were executed in a standardized, upright patient position with a total of 19 frequencies ranging from 1 kHz to 1 MHz. Waist circumference in centimeters was determined manually for each patient undergoing BIA.

### Statistical analyses

All statistical analyses were conducted using R 3.4.0 with RStudio 1.0.136, and figures were created using the ggplot2 package. The Pearson correlation coefficient was employed to assess the correlation between cervical and abdominal CT measurements and further to assess the correlation between these CT measurements and BIA results. The concordance correlation coefficient was used to evaluate intra- and interreader reliability. Interpretation of the concordance correlation coefficient was as follows: excellent agreement (> 0.8), good agreement (> 0.6), moderate agreement (> 0.4), and poor agreement (≤ 0.4). Otherwise, continuous variables are reported as mean ± standard deviation (SD). Statistical significance was set as *p* ≤ 0.05.

## Results

### Patient characteristics

Of 122 included patients (mean age 63 ± 16 years), 69 were male with a mean age of 64 ± 17 years and 53 were female with a mean age of 63 ± 15 years. As their underlying disease, 114 patients had malignant melanoma, 5 patients had a Merkel cell carcinoma, 2 patients had a squamous cell carcinoma, and one patient each had a dermal sarcoma and a cutaneous B cell lymphoma. In the subset of 30 patients for which the results of BIA were available, all patients were diagnosed with malignant melanoma. The average time interval between BIA and CT examination was 21 ± 16 days.

### Correlation of cervical and abdominal CT measurements

**Fig. 3:**
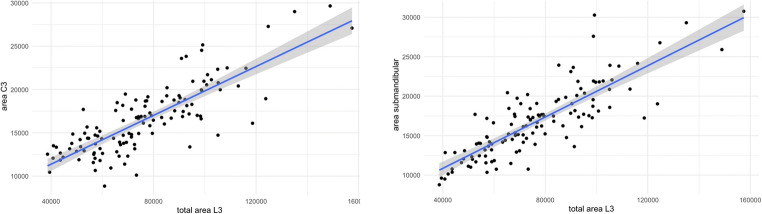
Correlations between the total cross-sectional submandibular area (on the left) and at the height of the third cervical vertebra (C3, on the right) and the total cross-sectional area at the height of the third lumbar vertebra (L3). All values are reported in mm^2^

The total cross-sectional area of the patient’s body at the submandibular level and at the height of C3 both showed a strong correlation with the total cross-sectional area at the height of L3 of *r* = 0.83 and *r* = 0.81, respectively (both *p* < 0.001; see Fig. 3). For the estimation of the total cross-sectional area at the height of L3, the following equations were obtained:

$$ \mathrm{Area}\ \mathrm{L}3=-756.3+4.68\times \mathrm{area}\ \mathrm{C}3 $$

$$ \mathrm{Area}\ \mathrm{L}3=5471.8+4.24\times \mathrm{submandibular}\ \mathrm{area}\ \mathrm{C}3 $$

**Fig. 4:**
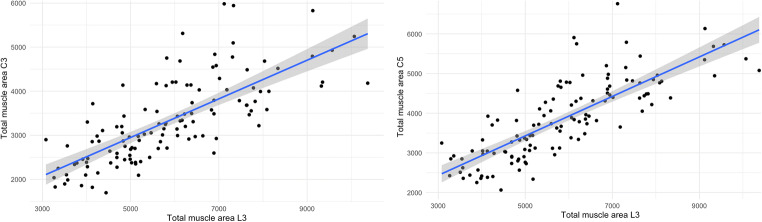
Correlations between the paraspinal muscle area at the height of the third (C3, on the left) and fifth (C5, on the right) cervical vertebrae and the total abdominal paraspinal muscle area at the height of the third lumbar vertebra (L3). All values are reported in mm^2^

Regarding the muscle areas, the total cervical paraspinal muscle area at the height of both C3 and C5 showed strong correlations with the total abdominal paraspinal muscle area of *r* = 0.74 and *r* = 0.8, respectively (both *p* < 0.001; see Fig. 4). For the estimation of muscle areas at the height of L3, the following equations were obtained:

$$ \mathrm{Muscle}\ \mathrm{area}\ \mathrm{L}3=1696+1.25\times \mathrm{muscle}\ \mathrm{area}\ \mathrm{C}3 $$

$$ \mathrm{Muscle}\ \mathrm{area}\ \mathrm{L}3=932.3+1.28\times \mathrm{muscle}\ \mathrm{area}\ \mathrm{C}5 $$

Mean values of the measured areas are presented in Table 2.

### Correlation of CT measurements and BIA

In the subset of 30 patients, for whom additional BIA information was available, both the total cross-sectional area of the patient’s body at the submandibular level and that at the height of C3 showed strong correlations with the total fat mass obtained from BIA (*r* = 0.82/*r* = 0.80; both *p* < 0.001) and the visceral fat mass (*r* = 0.92 and *r* = 0.90, both *p* < 0.001). Similarly, strong correlations between the total cross-sectional area of the patient’s body at the submandibular level and at the height of C3 and waist circumference were found (*r* = 0.92 and *r* = 0.86, both *p* < 0.001). Regarding the correlation between abdominal total cross-sectional area at the height of L3 and BIA measurements, strong correlations were found for total fat mass (*r* = 0.90, *p* < 0.001), for visceral fat mass (*r* = 0.90, *p* < 0.001), and for waist circumference (*r* = 0.94, *p* < 0.001).

Moderate to strong correlations of *r* = 0.44 and *r* = 0.72 were found between the total cervical paraspinal muscle area at the height C3 and C5 and muscle mass determined by BIA (*p* = 0.02 and *p* < 0.001). A correlation of *r* = 0.67 was found between the total abdominal paraspinal muscle area and the absolute muscle mass (*p* < 0.001). Detailed results are presented in Table 1 and ESM 1.

**Table 1:** Pearson correlation coefficients for correlations between cervical and abdominal CT measurements and between cervical CT measurements and bioelectrical impedance analysis (BIA) results

	Area L3	Muscle area L3	BIA total fat mass	BIA visceral fat mass	Waist circumference	BIA muscle mass
Area submandibular	0.83 *p < 0.001*		0.82 *p < 0.001*	0.92 *p < 0.001*	0.92 *p < 0.001*	
Area C3	0.81 *p < 0.001*		0.80 *p < 0.001*	0.90 *p < 0.001*	0.86 *p < 0.001*	
Muscle area C3		0.74 *p < 0.001*				0.44 *p = 0.02*
Muscle area C5		0.80 *p < 0.001*				0.72 *p < 0.001*

Both intra- and interreader reliability measurements showed excellent agreements, ranging from concordance correlation coefficients of 0.81 to 0.99 and from 0.82 to 0.99, respectively. Detailed results of intra- and interreader reliability measurements are provided in Table 2.

**Table 2:** Detailed mean values in mm^2^ and corresponding standard deviation of the performed CT measurements as well as concordance correlation coefficients (CCC) for intra- and interreader reliability measurements

	Mean	Standard deviation	CCC (intrareader)	CCC (interreader)
Total cross-sectional area L3	76,842.52	22,860.81	0.99	0.99
Muscle area L3	5858.23	1588.71	0.99	0.97
Total cross-sectional area submandibular	16,844.3	4479.12	0.96	0.82
Total cross-sectional area C3	16,571.29	3966.86	0.81	0.98
Muscle area C3	3325.53	940.16	0.95	0.93
Muscle area C5	3855.43	991.6	0.97	0.97

## Discussion

Sarcopenia, sarcopenic obesity, and loss of muscle mass correlate positively with adverse outcomes and mortality in a variety of medical conditions [3, 18–21]. While surrogate markers in abdominal CT imaging have been extensively analyzed, such markers are unavailable for examinations of the head and neck. This substantiates the necessity for reliable and easy-to-perform approaches in patients who solely receive head and neck CT examinations. In this study, we report markers that can be easily grasped from head and neck CT that correlate closely with surrogate markers established in abdominal imaging and hence may provide specific benefit in assessment of sarcopenia and sarcopenic obesity in patients with neurological disorders.

We found strong correlations between both the total cross-sectional areas at the level of C3 and directly submandibular and the total cross-sectional area at the level of L3. For the latter, strong correlations with both absolute fat mass and visceral fat mass have already been demonstrated [6, 22, 23]. Likewise, both total cross-sectional areas at the neck correlated strongly with the fat masses determined by BIA. Here, a slightly better correlation of the submandibular total cross-sectional area compared to the area at the level of C3 was observed for all parameters. Strong correlations were also found between muscle areas at C3 and C5 and the reference measurement at L3. Compared with the fat masses, slightly weaker correlations were found between the cervical muscle areas and the muscle mass determined by BIA; however, correlations were still predominantly strong. One likely explanation might be that in BIA all muscles (including extremities) are included in the measurement, while imaging-based assessment is limited to the torso. Fat, on the other hand, likely follows a more even distribution and hence shows a better correlation between the modalities. The variation of correlations might as well be impacted by the limited sample size of patients undergoing BIA. Lastly, while BIA is an accepted and established reference standard, it is known to be erroneous if not carried out according to a standardized patient preparation with regard to hydration state (which has been considered in our cohort) [24, 25]. Overall, in our view, the measurements of the submandibular total cross-sectional area as well as the measurement of the paraspinal muscle areas at the level of C5 appear to be suitable parameters to measure body composition in neck CT examinations. A possible explanation for the better correlation of muscle area at the C5 level with abdominal muscle area and actual muscle masses found in this study might be the different positioning of the patients during the CT examination. Head flexion or reclination as well as deviation in the horizontal may have a stronger effect further distally than close to the trunk at the level of C5 and thus cause a higher degree of variance.

These results differ to some extent from those of previous studies, which proposed measurements of muscle areas at the level of C3. For example, Jung et al found a moderate correlation between cross-sectional muscle areas at the height of C3 and L3 [15]. However, additional consideration of extra parameters (age, sex, and weight) allowed for a more accurate prediction of abdominal muscle areas [15]. Strong correlations were described by Swartz et al between 2D measurements of muscle areas at the level of C3 and L3 in their study [16]. However, the proposed measurements by Swartz et al were performed with prior consideration of density limits and using a dedicated software solution [16]. Overall, it can be argued that such special preparations or the need to consider additional clinical parameters limits the usability and implementation in daily routine. However, in most cases, this information can certainly be determined with reasonable effort and the creation of an algorithm or simple software that includes these factors should be straightforward. Furthermore, previous studies did not investigate whether measurements in neck CT might be used to predict patients’ fat masses, but only focused on determining muscle masses and/or areas.

In this respect, the present study differs from previous work as the described 2D ROI measurements are easy to perform in any PACS application and allow estimation of both muscle and fat masses. Furthermore, to our knowledge, this is the first study to compare 2D measurements on head and neck CT with a non-imaging reference standard.

The measurements we describe may facilitate an improved evaluation of the influence of body composition parameters in patients who undergo CT of the neck only, such as patients with suspected stroke. For example, data investigating a link between detailed parameters of body composition and outcome in patients with stroke is sparse [11].

This study has further limitations besides its retrospective design, which need to be mentioned. First, the proportion of patients for whom additional BIA data were available is limited. A larger fraction would be desirable but, unfortunately, was not available. In addition, there are several approaches to determine body composition in abdominal cross-sectional imaging. In the present study, validated and easy-to-perform 2D measurements on single CT slices were chosen as reference. Other methods, such as a voxel-by-voxel analysis of all muscle and adipose tissue, may provide different results. Recently, more and more AI-based approaches have been described to determine body composition. Therefore, it can be argued that it would be more useful to develop fully automated algorithms that can obtain body composition information in head and neck CT without manual measurements. Manual segmentations are inherently more susceptible to a higher intra- and interreader variability compared to fully automated methods. Nevertheless, we found excellent agreements, which underline the simplicity of the measurements. Several studies have demonstrated an influence of contrast media application on the determination of body composition, mostly showing a modest but significant increase in muscle areas after contrast media administration. Since the present study included only examinations of the neck and chest and abdomen, there is also a potential for bias due to the double administration of contrast medium [10, 26, 27]. Furthermore, it cannot be excluded with certainty that the underlying oncological disease of the included patients caused an influence on the correlations between abdominal and cervical measurements. Furthermore, the measurements presented in this study have not yet been evaluated for their potential to identify sarcopenic patients which might be in the scope of future studies. Lastly, the described method may have limitations in patients with abnormal fat distribution, for example, under cortisone treatment.

In conclusion, this study describes measurements that can be easily performed on single CT slices of head and neck CT and correlate very well with established measurements of body composition in abdominal CT examinations and results of BIA. Therefore, the reported measurements allow for a simple and reliable estimation of body composition in patients who receive a neck CT only and may substantiate their aptitude in future studies.

## Supplementary Information

ESM 1 The upper row shows correlations between muscle mass determined by bioelectrical impedance analysis (BIA) and the paraspinal muscle area at the height of the third cervical vertebra (C3, upper row on the left) and at the height of the fifth cervical vertebra (C3, upper row on the right). The bottom row depicts correlations between the total fat mass determined by BIA and the total cross-sectional muscle area at the height of the third cervical vertebra (C3, bottom row on the left) and at the submandibular level (bottom row on the right). BIA values are reported in kg, CT measurements in mm^2^ (PNG 2252 kb)

## Abbreviations

BIA: Bioelectrical impedance analysis
C3: Third cervical vertebra
C5: Fifth cervical vertebra
DEXA: Dual-energy X-ray absorptiometry
L3: Third lumbar vertebra

## Declarations

### Guarantor

The scientific guarantor of this publication is Nils Große Hokamp.

### Statistics and biometry

No complex statistical methods were necessary for this paper.

### Methodology

• retrospective

• diagnostic or prognostic study

• performed at one institution
