# Supplementary figures and images for: Two-dimensional CT measurements enable assessment of body composition on head and neck CT
Source: Eur Radiol. 2022 Apr 7;32(9):6427–34. doi: 10.1007/s00330-022-08773-9 (PMC9381610; doi:10.1007/s00330-022-08773-9)

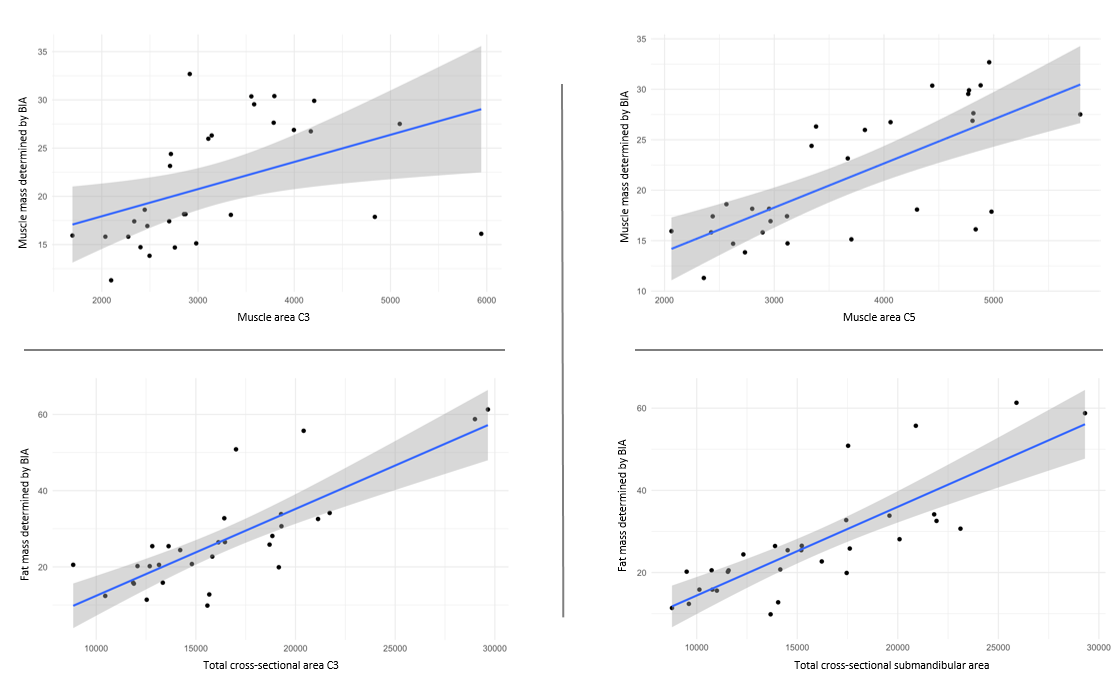

Supplement: Supplementary file 1 — The upper row shows correlations between muscle mass determined by bioelectrical impedance analysis (BIA) and the paraspinal muscle area at the height of the third cervical vertebra (C3, upper row on the left) and at the height of the fifth cervical vertebra (C3, upper row on the right). The bottom row depicts correlations between the total fat mass determined by BIA and the total cross-sectional muscle area at the height of the third cervical vertebra (C3, bottom row on the left) and at the submandibular level (bottom row on the right). BIA values are reported in kg, CT measurements in mm2 (PNG 2252 kb) [file 330_2022_8773_MOESM1_ESM.png]
